# Supplementary material for: Hiding in Plain Sight: Characterization of Aeromonas Species Isolated from a Recreational Estuary Reveals the Carriage and Putative Dissemination of Resistance Genes
Source: Antibiotics (Basel). 2023 Jan 4;12(1):84. doi: 10.3390/antibiotics12010084 (PMC9854640; doi:10.3390/antibiotics12010084)
Supplement: Supplementary file 1 [file antibiotics-12-00084-s001.zip › antibiotics-2029274-supplementary.pdf]

antibiotics

Supplementary Materials

Communication

Hiding in plain sight: characterization of *Aeromonas* species isolated from a recreational estuary reveals the carriage and putative dissemination of resistance genes

Anna Luiza Bauer Canellas[1], Bruno Francesco Rodrigues de Oliveira[2], Marinella Silva Laport[1]*

[1] Laboratório de Bacteriologia Molecular e Marinha, Instituto de Microbiologia Paulo de Góes, Universidade Federal do Rio de Janeiro (UFRJ), Av. Carlos Chagas Filho, 373, Cidade Universitária, 21941-902 Rio de Janeiro, Brazil; annaluiza@micro.ufrj.br
[2] Departamento de Microbiologia e Parasitologia, Instituto Biomédico, Universidade Federal Fluminense, 24210-130 Niterói, RJ, Brazil; bfroliveira@id.uff.br
* Correspondence: marinella@micro.ufrj.br

Index

Figure S1 Guanabara Bay sampling sites (green dots). Site 1 is located in the proximity of the Atlantic Ocean and is one of the least impacted sites. Site 7 is considered the intermediate site and site 34 is located in the inner part of the estuary, being considered one of the most ecologically impacted sites. Red markers indicate wastewater treatment plants……………………………………..……………………….2

Table S1 Primer sequences, cycling conditions and amplicon sizes of the antimicrobial resistance genes and the class 1 integron-integrase gene investigated in this study………3

Table S2 Primer sequences, cycling conditions and amplicon sizes of the heavy metal resistance genes and the class 1 integron-integrase gene investigated in this study……...4

References…………………………………………………………………………….5

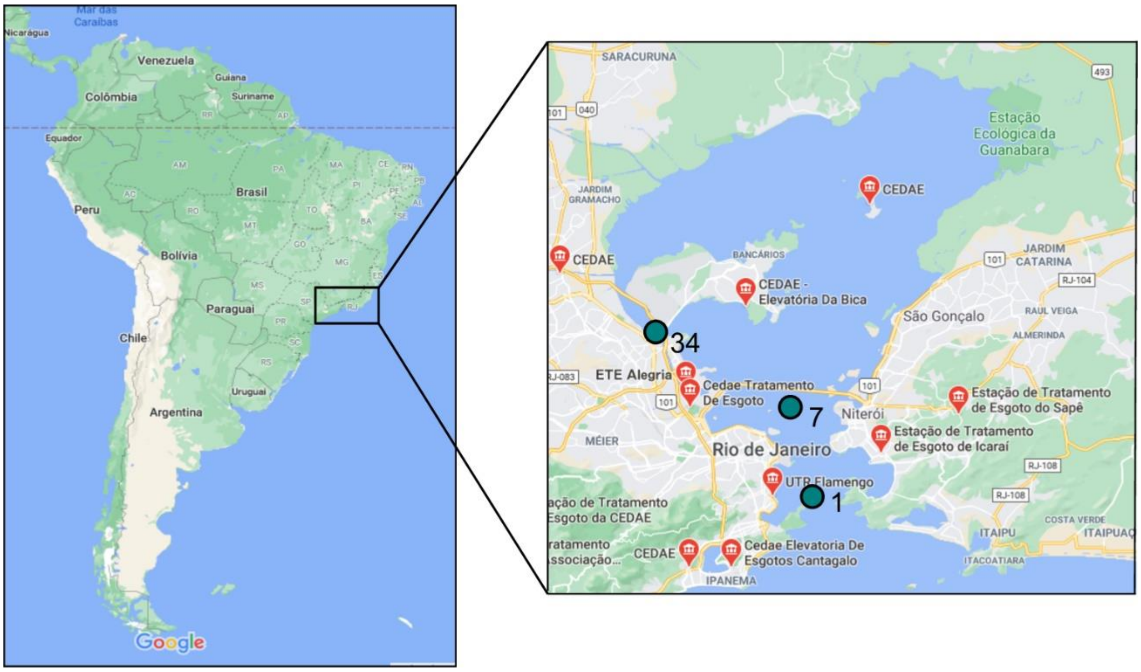

Figure S1 Guanabara Bay sampling sites (green dots). Site 1 is located in the proximity of the Atlantic Ocean and is one of the least impacted sites. Site 7 is considered the intermediate site and site 34 is located in the inner part of the estuary, being considered one of the most ecologically impacted sites. Red markers indicate wastewater treatment plants.

Table S1 Primer sequences, cycling conditions and amplicon sizes of the antimicrobial resistance genes investigated in this study.

Resistance gene	Primer	Sequence	Cycling conditions	Amplicon size (bp)	References
bla$_{GES}$	mGESF	5'-AGCAGCTCAGATCGGTGTTG-3'	95ºC/10' + 30 cycles of 95ºC/30", 54ºC/30", 72ºC/45" and final extension of 72ºC/10'	750	[1,2]
	mGESR	5'-CCGTGCTCAGGATGAGTTG-3'			
bla$_{SHV}$	mSHVF	5'-CTTGACCGCTGGGAAACGG-3'		200	
	mSHVR	5'-AGCACGGAGCGGATCAACGG-3'			
bla$_{TEM}$	mTEMF	5'-CCCTTATTCCCTTTYTTGCGG-3'		650	
	mTEMR	5'-AACCAGCCAGCCWGAAGG-3'			
bla$_{CTX-M-1,2}$	mCTX-1,2F	5'-ATGTGCAGYACCAGTAA-3'		512	
	mCTX-1,2R	5'-CGCTGCCGGTTTTATCSCCC-3'			
bla$_{CTX-M-8}$	mCTX-8F	5'-AACRCRCAGACGCTCTAC-3'		333	
	mCTX-8R	5'-TCGAGCCGGAASGTGTYAT-3'			
bla$_{CTX-M-14}$	mCTX-14F	5'-GGTGACAAAGAGARTGCAACGGAT-3'		876	
	mCTX-14R	5'-TTACAGCCCTTCGGCGATGA-3'			
bla$_{KPC}$	KPC1F	5'-GCTACACCTAGCTCCACCTTC-3'	94ºC/5' + 30 cycles of 94ºC/1'; 61ºC/30''; 72ºC/1' and final extension of 72ºC/5'	950	[3]
	KPC1R	5'- TGGAGGGCCAATAGATGATT-3'			
mcr-1	mcr1-mtpF	5'-ATGCCAGTTTCTTTCGCGTG-3'	94ºC/4' + 30 cycles of 94ºC/5''; 59ºC/20''	502	[4]
	mcr1-mtpR	5'-TCGGCAAATTGCGCTTTTGGC-3'			
mcr-2	mcr2-mtpF	5'-GATGGCGGTCTATCCTGTAT-3'		379	
	mcr2-mtpR	5'-AAGGCTGACACCCCATGTCAT-3'			
mcr-3	mcr3-mtpF	5'-ACCAGTAAATCTGGTGGCGT-3'		296	
	mcr3-mtpR	5'-AGGACAACCTCGTCATAGCA-3'			
mcr-4	mcr4-mtpF	5'-TTGCAGACGCCCATGGAATA-3'		207	
	mcr4-mtpR	5'-GCCGCATGAGCTAGTATCGT-3'			
mcr-5	mcr5-mtpF	5'-GGACGCGACTCCCTAACTTC-3'		608	
	mcr5-mtpR	5'-ACAACCAGTACGAGAGCACG -3'			

Table S2 Primer sequences, cycling conditions and amplicon sizes of the heavy metal resistance genes and the class 1 integron-integrase gene investigated in this study.

Resistance gene	Primer	Sequence	Cycling conditions	Amplicon size (bp)	References
merA	merA-F<br>merA-R	5'-GTGCCGTCCAAGATCATGAT-3'<br>5'-TAGCCYACRGTSGCSACYTG-3'	94ºC/4 min + 30 ciclos de 94ºC/1 min; 57ºC/1 min; 72ºC/1 min e extensão final a 72ºC/8 min	933	[5]
	A1F<br>A5R	5'-ACCATCGGCGGCACCTGCGT-3'<br>5'-ACCATCGTCAGGTAGGGGACCAA-3'	95ºC/5 min + 30 ciclos de 94ºC/15 s; 63ºC/30 s; 68ºC/1 min e extensão final a 68ºC/4 min	1226	[6]
merB	IA34F<br>IA35R	5'-TTGGATCCATGAAGCTCGCCCCAT-3'<br>5'-TTGGTACCCTAGATGACATGACAT-3'	95°C/ 5 min + 35 ciclos de 95°C/ 1 min; 61 ºC/1 min; 72 ºC/ 1 min e extensão final a 72°C/ 10 min	639	[7]
cusB	czcB-I<br>czcB-II	5'-ATGAAAAAAATCGCGCTT-3'<br>5'- ATGCGCATGGGTAGCACT-3'	94ºC/5 min + 35 ciclos de 94ºC/30 s; 51ºC/45 s; 72ºC/1 min e extensão final a 72ºC/10 min	1200	[8]
copA	cad1<br>cad2	5'-CAAAYTGYGCRGGHAARTTYGA-3'<br>5'-AACTAATGCACAAGGACA-3'	94ºC/5 min + 30 ciclos de 94ºC/1 min; 60ºC/1 min e 30 s; 72ºC/2 min e extensão final a 72ºC/5 min	1058	[9]
pbrA	pbrA-N<br>pbrA-C	5'-ATGAGCGAATGTGGCTCGAAG-3'<br>5'-TCATCGACGCAACAGCCTCAA-3'	96ºC/5 min + 39 ciclos de 96ºC/1 min; 58ºC/1 min; 72ºC/3 min e extensão final a 72ºC/10 min	2400	[10]
intI1	INT1-F<br>INT1-R	5'-GGCTTCGTGATGCCTGCTT-3'<br>5'-CATTCCTGGCCGTGGTTCT-3'	94ºC/10 min + 35 ciclos de 98ºC/30 s; 60ºC/30 s; 72ºC/30 s e extensão final a 72ºC/10 min	146	[11]

References

[1] Campana, E.H.; Xavier, D.E.; Petrolini, F.V.; Cordeiro-Moura, J.R.; Araujo, M.R.; Gales, A.C. Carbapenem-resistant and cephalosporin-susceptible: a worrisome phenotype among *Pseudomonas aeruginosa* clinical isolates in Brazil. *Braz. J. Infect. Dis.* **2017**, *21*(1), 57-62.

[2] da Costa, W.F.; Giambiagi-deMarval, M.; Laport, M.S. *Shewanella* harboring antimicrobial and copper resistance genes in sea urchins (*Paracentrotus lividus*) from the Crozon peninsula (Brittany, France). *Infect. Genet. Evol.* **2020**, *85*, 104437.

[3] Hossain, A.; Ferraro, M.J.; Pino, R.; Dew III, R.B.; Moland, E.S.; Lockhart, T.J.; Thomson, K.S.; Goering, R.V.; Hanson, N.D. Plasmid-mediated carbapenem-hydrolyzing enzyme KPC-2 in an *Enterobacter* sp. *Antimicrob. Agents Chemother.* **2004**, *48*(11), 4438-4440.

[4] Lescat, M.; Poirel, L.; Nordmann, P. Rapid multiplex polymerase chain reaction for detection of *mcr-1* to *mcr-5* genes. *Diagn. Microbiol. Infect. Dis.* **2018**, *92*(4), 267-269.

[5] Deredjian, A.; Colinon, C.; Brothier, E.; Favre-Bonté, S.; Cournoyer, B.; Nazaret, S. Antibiotic and metal resistance among hospital and outdoor strain of *Pseudomonas aeruginosa*. *Res. Microbiol*. **2011**, 162, 689-700.

[6] De, J.; Ramaiah, N.; Vardanyan, L. Detoxification of toxic heavy metals by marine bacteria highly resistant to mercury. *Mar. Biotechnol.* (NY). **2008**, *10*, 471-477

[7] Murtaza, I.; Dutt, A.; Mushtaq, D.; Ali, A. Molecular cloning and genetic analysis of functional *merB* gene from Indian isolates of *Escherichia coli*. *Curr. Microbiol*. **2005** *51*(5), 297-302.

[8] Khan, Z.; Nisar, M.A.; Hussain, S.Z.; Arshad, M.N.; Rehman, A. Cadmium resistance mechanism in *Escherichia coli* P4 and its potential use to bioremediate environmental cadmium. *Appl. Microbiol. Biotechnol*. **2015**, *99*, 10745-10757.

[9] Oger, C.; Bertheb, T.; Quilleta, L.; Barraya, S.; Chiffoleauc, J.F.; Petit, F. Estimation of the abundance of the cadmium resistance gene *cadA* in microbial communities in polluted estuary water. *Res. Microbiol.* **2001**, *152*, 671-678.

[10] Borremans, B.; Hobman, J.L.; Provoost, A.; Brown, N.L.; van-Der-Lelie, D. Cloning and functional analysis of the pbr lead resistance determinant of *Ralstonia metallidurans* CH34. *J. Bacteriol.* **2001**, *183*, 5651-5658.

[11] Luo, Y.I.; Mao, D.; Rysz, M.; Zhou, Q.; Zhang, H.; Xu, L.; JJ Alvarez, P. Trends in antibiotic resistance genes occurrence in the Haihe River, China. *Environ. Sci. Technol*. **2010**, *44*(19), 7220-7225.
